# Self-Configuring Indoor Localization Based on Low-Cost Ultrasonic Range Sensors

**DOI:** 10.3390/s141018728

**Published:** 2014-10-10

**Authors:** Can Basaran, Jong-Wan Yoon, Sang Hyuk Son, Taejoon Park

**Affiliations:** 1 Northern Cyprus Campus, Middle East Technical University, Mersin 10, Turkey; E-Mail: basaran@metu.edu.tr; 2 Department of Information and Communication Engineering, Daegu Gyeongbuk Institute of Science and Technology (DGIST), 333 Techno Jungang-Daero, Hyeonpung-Myeon, Dalseong-Gun, Daegu 711-873, Korea; E-Mails: jwyoon@dgist.ac.kr (J.-W.Y.); son@dgist.ac.kr (S.H.S.)

**Keywords:** self-configuration, indoor localization, device-free localization, ultrasonic sensors

## Abstract

In smart environments, target tracking is an essential service used by numerous applications from activity recognition to personalized infotaintment. The target tracking relies on sensors with known locations to estimate and keep track of the path taken by the target, and hence, it is crucial to have an accurate map of such sensors. However, the need for manually entering their locations after deployment and expecting them to remain fixed, significantly limits the usability of target tracking. To remedy this drawback, we present a self-configuring and device-free localization protocol based on genetic algorithms that autonomously identifies the geographic topology of a network of ultrasonic range sensors as well as automatically detects any change in the established network structure in less than a minute and generates a new map within seconds. The proposed protocol significantly reduces hardware and deployment costs thanks to the use of low-cost off-the-shelf sensors with no manual configuration. Experiments on two real testbeds of different sizes show that the proposed protocol achieves an error of 7.16∼17.53 cm in topology mapping, while also tracking a mobile target with an average error of 11.71∼18.43 cm and detecting displacements of 1.41∼3.16 m in approximately 30 s.

## 1. Introduction

Target tracking is a process of continuously recording the locations of a target, e.g., a human, in time. In smart environments, this is an essential service because most of the user interactions are through location-aware activities [1]. Health-care systems report the location of a user to medical professionals in the event of an emergency; activity recognition systems use the rooms that a user has occupied and the objects s/he has used at a certain point of time in order to infer the context; and infotainment systems use similar information to provide a user with personal feeds as s/he moves inside the environment. These interaction models, tightly coupled with location awareness, mean that the smart space experience is shaped by the existence and accuracy of a target tracking system.

Target tracking solutions can be broadly categorized into either *intrusive* or *non-intrusive* systems. The former uses a dedicated device (target) carried by a user to send messages, e.g., Wi-Fi signals, to the system, while the latter or a *device-free* system tries to solve the problem without relying on such a device [2]. The identity of a user is readily available in the intrusive system since this device can contain a unique identifier. By contrast, in the non-intrusive system, a user is identified by analyzing sensor readings such as weight and/or height measurements, or by face recognition at certain checkpoints in the environment [3]. Once the user is identified, the system may start sampling the location of the user regularly in order to construct a *track*.

Whether intrusive or not, the target should be located based on its distance to some reference points that have known locations. Therefore, the system also needs to preconfigure or determine the locations of the reference points, *i.e., landmarks*; this process can be rehashed into a sensor localization problem that determines relative or absolute locations of sensor nodes in a sensor network [4]. However, the problem is not trivial when applied to the smart environments since GPS devices cannot be used indoors and also the walls and furniture cause interference and multipath effects, hindering the accuracy of distance measurements [5]. Moreover, smart home sensor localization solutions [6–8] generally depend on manual mapping of landmarks, but manually defining their locations is undesirable as it complicates the deployment and makes the system fragile. For example, if somehow one sensor is displaced after mapping, the system will fail to operate and will require manual reconfiguration.

Motivated by these limitations, we aim to design the tracking system to work out of the box as illustrated in the following deployment scenario. A user simply ‘puts’ sensors at convenient locations and turns them on. Then the sensors cooperatively pass through a *self-configuration* phase, in which they collect data on users’ movements across their field-of-view for a short period of time and transform themselves into landmarks for target tracking by having their relative locations calculated and memorized. The system then switches to a *tracking phase*, in which the landmarks calculate the locations of users in real-time as well as monitor their surroundings to detect any sensor displacements and fix the sensor topology if detecting changes.

To realize this scenario, we propose a *self-configuring* and *low-cost* localization protocol for smart environments using cheap off-the-shelf distance sensors such as ultrasonic rangers [9]. The proposed protocol is capable of facilitating *device-free* indoor tracking of users with zero prior knowledge of the geographic topology while also allowing hassle-free deployment without the need for manual configuration. As opposed to traditional localization protocols that rely on estimated distances between sensor nodes, the proposed protocol works on concurrent distance measurements to a mobile target or a user. The key idea of the proposed protocol is to exploit the mobility of the user for accurate localization and tracking. To do so, our protocol applies a Genetic Algorithm (GA) for identifying the locations of sensors on a 2-dimensional space, which yields sufficiently accurate results compared to outdoor environments. The located sensors are then used as landmarks for indoor target tracking. Moreover, our protocol effectively handles the events of sensor displacement, which is common in our user-friendly deployment scenario, by employing a lightweight scheme for detecting such events in real time and recovering from changes to sensor locations. Our experiments on two real testbeds of different sizes, *i.e.*, 2 × 3 m^2^ and 4 × 6 m^2^, indicate that the proposed protocol can achieve an error of 7.16 to 17.53 cm in identifying landmark locations while also tracking a mobile target with an average error of 11.71 to 18.43 cm. Moreover, we observe the proposed protocol is capable of detecting and handling displacements of magnitude, 1.41∼3.16 m in approximately 30 s.

The rest of the paper is organized as follows. In Section 2 we go over the literature and give a brief summary of the related work. In Section 3 we briefly go over the sensor hardware used in our implementation and discuss its limitations. In Sections 4 and 5, we describe our self-configuring localization and tracking algorithms in details. Based on the need for easy deployment of sensing infrastructure, we design a self-configuring, device-free, and cost-effective localization protocol that uses distance measurements to the target for accurate localization among landmark sensors, and further extend the protocol to automatically identify the displacement of landmarks by reusing the localization algorithm. We further develop a heuristic-based target tracking system based on the genetic algorithm by employing the thus-designed self-configuring protocol, and show that our system is robust to measurement errors in contrast to the existing algorithms, e.g., those based on mathematical formulations, because it finds the best solution that fits the measurements. In Section 6 we present the experimental setup and provide results of comprehensive experiments conducted on the real testbed. We demonstrate that the protocol significantly reduces the system complexity and deployment cost as well as achieves high level of accuracy in tracking targets over a broad range of network layouts. Finally, we discuss possible future directions and conclude this paper in Section 7.

## 2. Related Work

Device-aided indoor tracking systems benefit from GPS devices, cell phones, or RFID tags [10–13]. For instance, the algorithm in [14] describes an anchor-free indoor localization technique based on the strength of Wi-Fi signals. However, this algorithm relies on infrequent GPS checkpoints and requires users to carry a Wi-Fi receiver. These systems typically use collaborating devices carried by the users, and may be most suitable for in-building localization/tracking systems, while in a smart home environment devices create significant inconvenience for the users. A method for locating humans within a room using cameras has been proposed, but using cameras is undesirable in a smart home environment due to privacy issues [15]. This is especially true for bedrooms and bathrooms where people feel an invasion of privacy in the presence of visual sensors. SCPL is a device-free tracking system which uses radio signals to track multiple people [16]. However, the system requires a known floor plan of the environment and this considerably complicates the deployment process.

The use of GA for sensor network localization is not novel by itself. In fact, localization is an interesting application of GA as shown in [8,17–20]. One of the previous GA-based algorithms, GA-Loc assumes that distances between one-hop neighbors are known and tries to minimize the difference between measured distances and those that are obtained by the estimated locations [19]. While this algorithm does not necessitate nodes with known locations, it uses inter-node distances and obtaining this information indoors requires complex hardware. Mark *et al.* [21] introduced a two-phase GA-based algorithm using distances between one-hop neighbors. The first phase calculates locations of nodes that have three immediate anchor neighbors via trilateration. The second phase includes all other nodes and uses GA to minimize difference between known and estimated distances between nodes. The work on the use of GA-based systems in target tracking is scarce in the literature. The main focus of existing work is associating sensor readings with targets in multi-target tracking systems [22,23]. However, our proposed system does not focus on multi-target tracking.

While none of these algorithms cannot simultaneously meet the requirements of anchor-free, device-free, low-cost, and high-accuracy localization, our proposed algorithm successfully addresses these requirements by associating GA with human mobility.

## 3. Sensor Hardware and Related Challenges

### 3.1. Sensor Hardware

We aim to realize a cost-effective localization solution by using off-the-shelf ultrasonic ping sensors for distance measurement [2,24]. A ping sensor, having a transducer and a receiver, measures the distance to an object by emitting a short ultrasonic 40 kHz burst and then calculating the *time difference of arrival* (TDOA) of the reflected wave with respect to the original burst. Since the echo is reflected from the object of interest, the distance can be calculated by considering the speed of sound and the TDOA. Note that the speed of sound is affected by both temperature and humidity. While the latter (humidity) does not significantly impact the sound propagation in environments with normal air pressure, the former (temperature) must be considered in calculating the speed of sound [25,26]. This sensor has a 40° beam angle, and can sense objects within a range of 2∼300 cm. One issue with this type of sensors is how to determine if the reflecting surface is a part of the environment such as a wall or furniture, or a mobile target. Another problem is their vulnerability to interference from other ultrasonic sources; for instance, the receiver of one sensor may sense the signal from the transducer of another sensor when concurrent readings are taken from different sensors. A similar problem occurs if a sensor picks up an echo of a previous signal still residing in the room. In such cases the readings are meaningless and should be discarded. One way to prevent these problems is to introduce a delay, ε, between subsequent sensor readings [9]. In our implementation, two measurements are at least 0.2 s apart, *i.e.*, ∊ = 0.2.

### 3.2. Formation of Input Database

The distance measurements are accumulated in the input database 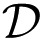, in which each entry *d_tk_* is the distance of the target to sensor *k* at time *t* where *k* = 1, ⋯, *K*. Note that a typical value of *K* is 4. We define a row *d_t_* = {*d_t_*_1_ ⋯ *d_tK_*} of 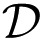 to be a *problem instance*. If an actual geographic map of sensors is already known, we can use three *valid* (*i.e*., noise-free) elements in *d_t_* to calculate the location of the target at time *t* using trilateration [27]. Solving a problem instance to get the location requires that all elements in *d_t_* are measurements to the same reference point. However, since we consider a mobile target as the point of reference, the sensing delay ε between consecutive measurements becomes a major source of error. In case the mobile target moves between two measurements during the construction of *d_t_*, the assumption of having a common reference point across *d_t_* breaks. Furthermore, as the number of elements in *d_t_, i.e.*, |*d_t_*|, increases, the problem gets worse since the maximum time difference between any pair of measurements can be as large as ∊ × (|*d_t_*| − 1) and a possible shift in the position of the reference point between measurements in *d_t_* can be significant. Another source of error is the inaccuracy of distance measurements. Although ultrasonic sensors have an error range less then ±5 cm, the method of measurement is vulnerable to multipath effects as explained earlier.

**Figure 1.**
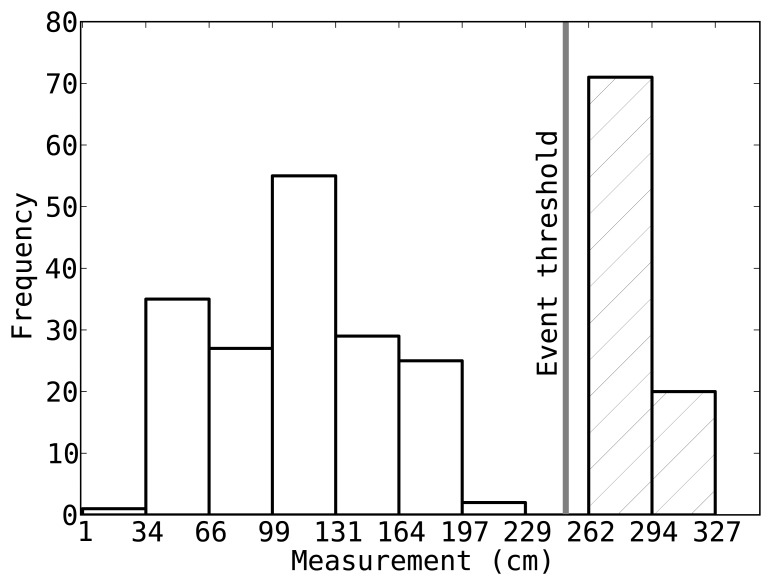
To remove outliers, each sensor marks the sensor reading higher than the event threshold (60% of the total readings) as invalid.

To deal with the above difficulties, we use a two-step approach to remove noise from 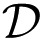: The first step erases non-event measurements (outliers) while the second step filters out low-quality measurements. When the mobile target enters into the line-of-sight, meaning that an event occurs, the sensor records a smaller TDOA value compared to the samples taken when the field-of-view is clear [2]. The first step exploits this property and analyzes the sensor readings by setting an *event threshold* to 60% of the readings. Hence, a reading less then this event threshold fires an event and is inserted into 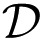, otherwise, the element corresponding to this sensor is marked as *invalid*. Figure 1 shows the event threshold for one of the sensors in our testbed along with the histogram of measurements recorded by that sensor. We note this simple method removes more than 99% of the non-event data from 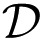 across all sensors.

**Figure 2.**
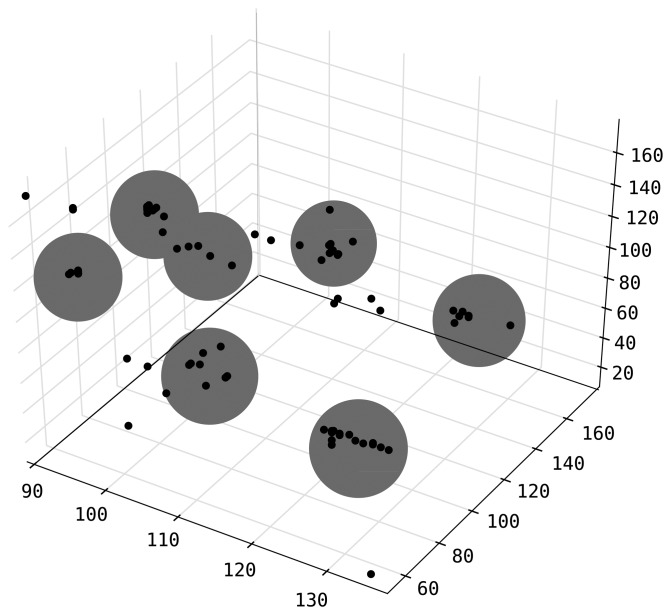
An example DBSCAN clustering of distance measurements from 3 sensors.

In the second step of noise removal, the rows of 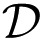 are clustered using a DBSCAN algorithm [28]. Interpreting *d_t_* as the *K*-dimensional coordinates of a target, DBSCAN clusters together rows that are in 10 cm proximity of each other in this *K*-dimensional space. Any cluster with less than 10 members is discarded. After this step, we can make sure that each row in 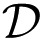 is an accurate observation of the same target. An example clustering result for measurements from 3 sensors is given in Figure 2 where dark points are readings and gray large dots mark cluster boundaries. We tested this filtering mechanism by collecting distance measurements to predetermined checkpoints while visiting the checkpoints regularly in an otherwise random walk fashion. We verified the system can identify outlier observations and filter them out.

Although clustering on 10 cm neighborhood, and using 10 members as the density threshold may seem arbitrary, the fixed sensing range and error characteristics of the hardware make this configuration generally applicable. This approach also handles errors due to the mobility of the target because clustered rows of 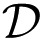 correspond to events in which the target is slow or stationary.

## 4. Self-Configuring Localization for Landmarks

In this section, we present a GA-based algorithm, called *Self-configuring Localization for Landmarks* (SeLL), to determine initially unknown locations of ultrasonic range sensors that will serve as landmarks for target tracking. SeLL works on a cluster of *K* sensors. Given an input database 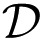 of distances, SeLL tries to find the actual locations of *K* sensors, *L* = {*l*_1_, *l*_2_, …, *l_K_*} by exploiting the movement of mobile target. As mentioned earlier, each row *d_t_* of 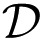 is a set of measurements to the same reference point obtained by *K* sensors. In fact these reference points associated with rows of 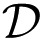 are snapshots of the mobile target’s trajectory. SeLL thus searches for a set of feasible locations, *L̂* = {*l̂*_1_, *l̂*_2_, …, *l̂_K_*} that would yield a solution for each and every reference point in 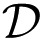, based on the assumption that a solution for all of the reference points can be found if and only if *L* = *L̂*. Once the relative locations of sensors in each cluster is found, these clusters are aggregated into a complete topology that includes all of the sensors in the environment.

As shown in Algorithm 1, SeLL starts with a set of z randomly generated estimations, *G* = {*L̂*_1_, … , *L̂_z_*}. In GA terms, G is the *population* and each *L̂_i_* = {*l̂_i_*_1_, *l̂_i_*_2_, …, *l̂_ik_*} is an *individual* that estimates the locations of sensors where *l̂_ik_* = {*x̂_ik_, ŷ_ik_*} is a point on a 2-dimensional plane representing the location of sensor *k* in *L̂_t_*. To calculate the location of the reference point based on *L̂_i_*, let us define *C_ik_* to be a circle centered at *l̂_ik_* with a radius of *d_tk_*, where *d_tk_* is the measured distance from *l̂_ik_* to the reference point. The following 3 steps constitute the main loop of the SeLL algorithm.


**Algorithm 1:** A self-configuring algorithm to determine locations of landmarks**Function** GA (*Max_gen_, R_x_, R_m_, z*) **begin** Create population *G* of *z* random individuals; *generation* = 0; **while**
*generation* < *Max_gen_*
**do**  Calculate fitness of all individuals;  *G′* = Ø;  **while** |*G*| < *z*
**do**   Select two parents, *p*1 and *p*2 from *G*;   Perform crossover with rate *R_x_*;   Perform mutation with rate *R_m_*;   Insert the offspring to *G′*   **end**   *G* = *G′*;  *generation* = *generation* + 1;  **end**  **return**
*the best individual*;**end**

**Step 1. Quality Estimation:** The quality of an individual *L̂_i_* is calculated using a *Distance* function which computes a value proportional to the dissimilarity between *L̂_i_* and *L*, or to the success of finding consistent solutions for 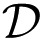. The GA algorithm tries to minimize the *Distance* in order to find the best *L̂_i_* that is closest to *L*. In other words, the *Distance* function associates a non-negative penalty value for a given estimation of the geographic layout of locations, *L̂_i_*.

**Figure 3.**
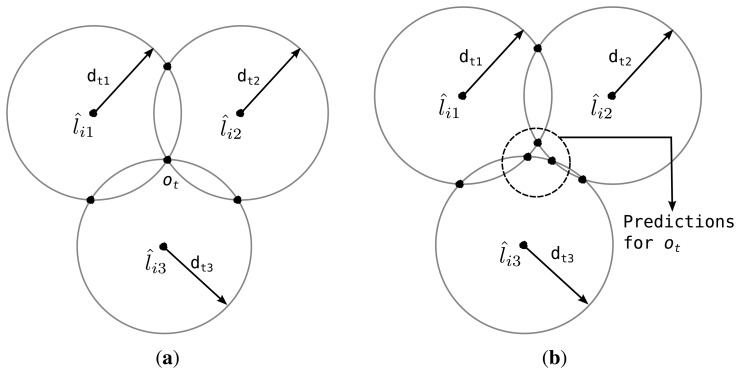
Calculation of the prediction error *e_it_*. (**a**) Perfect prediction; (**b**) Close prediction.

As noted earlier, only three elements in *d_t_* are sufficient for calculating the location of the reference point via trilateration [29]. Hence, let us first consider the case of having three location estimates in *L̂_i_*, say *l̂_i_*_1_, *l̂_i_*_2_ and *l̂_i_*_3_. SeLL forms three circles (*C_i_*_1_, *C_i_*_2_ and *C_i_*_3_) and finds two points of intersection for each of two-circle combinations of three circles, resulting in six intersection points (Figure 3). Among them, three points, one from each two-circle pair, that are closer to one another are selected. Then the reference point is within the triangle defined by these three points. With perfect inputs these three points overlap and the location of the reference point can be calculated with point accuracy, whereas the surface area of the estimation triangle increases as the error increases. Next, when the size of *L̂_i_* is greater than 3, the redundancy in *d_t_* can be used to enhance the quality of *L̂_i_*. That is, the location of the target is calculated with all combinations of two-circle pairs, {(*C_ik_, C_im_*) | *k* ≠ *m*} and corresponding distance measurements, {(*d_tk_, d_tm_*) | *k* ≠ *m*} by using the intersection of two circles.

The location of the reference point, although unknown, is the same across *d_t_*, and hence, we define a *prediction error* at time *t*, denoted by *e_it_*, as the aggregate difference between calculated locations of the reference point from the circle pairs. The Distance of *L̂_i_* is then given by the sum of all *e_it_*’s over the rows of 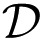. In Figure 3, *o_t_* denotes the actual location of the target at time *t*. As illustrated in Figure3a, *e_it_* = 0 if *L̂_i_* is the global minimum solution and there is no error in the distance measurements. However, the predictions for *o_t_* do not overlap for an inferior *L̂_i_* and *e_it_* > 0 as shown in Figure3b. The pseudo code for a simplified *Distance* function is given in Algorithm 2.


**Algorithm 2:** Simplified *Distance* function**Function**
*Distance*(*L̂_i_*, 

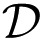
) **begin** *Distance* = 0; **foreach**
*d_t_* ∈ *D*
**do**  *O*′ = {};  **foreach**
*l̂_ik_* ∈ *L̂_i_ and l̂_im_* ∈ *L̂_i_, k* ≠ *m*
**do**   *C_ik_* = circle with radius *d_tk_* centered at *l̂_ik_*;   *C_im_* = circle with radius *d_tm_* centered at *l̂_im_*;   *o*_1_, *o*_2_ = IntersectionPoints(*L̂_i_,C_ik_, C_im_*);   Add *o*_1_, *o*_2_ to *O*′;  **end**  *O* = points from *O*′ that are closest to each other;  *e_it_* = Σ *pairwiseDistances*(*O*);  *Distance* = *Distance* + *e_it_*; **end****end**

**Figure 4.**
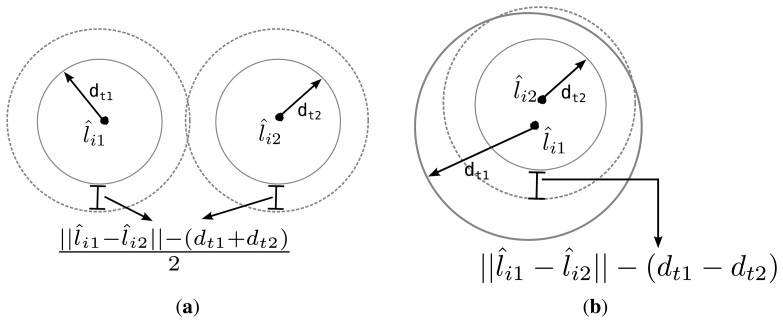
*Distance* function handling no-solution cases: when no intersection points are found, circles are extended to the dashed versions. (**a**) Case 1; (**b**) Case 2.

We further develop an algorithm for calculating the intersection points to deal with the cases in which some circle pairs do not overlap. We are concerned with the quality of *L̂_i_, i.e.*, it’s ability to solve *d_t_*, rather than the actual measurement error, the main source of which is the prediction error. To calculate the intersection points of two circles *C_ik_* and *C_im_*, we first compute a Euclidean distance between the centers of the circles as $∥{\hat{l}}_{ik}-{\hat{l}}_{im}∥=\sqrt{{\left({\hat{x}}_{ik}-{\hat{x}}_{im}\right)}^{2}+{\left({\hat{y}}_{ik}-{\hat{y}}_{im}\right)}^{2}}$. Depending on the Euclidean distance, three exceptional cases may arise: (1) if ∥*l̂_ik_* − *l̂_im_*∥ > *d_tk_* + *d_tm_* then *C_ik_* and *C_im_* are separate and there is no intersection; (2) if ∥*l̂_ik_* − *l̂_im_*∥ < |*d_tk_* + *d_tm_*|, then one circle is contained within the other and there is no intersection; and finally, (3) if ∥*l̂_ik_* − *l̂_im_*∥ = 0, *d_tk_* = *d_tm_* then the two circles coincide and there are infinite number of intersections. Note that, even though two candidate estimations *L̂_i_* and *L̂_j_* may both fall into the first case and yield no intersection, it is desirable that the *Distance* function assigns a lower rank for the candidate which is closer to the real topology *L*. Notably, simply assigning a constant large penalty to infeasible estimates results in poor performance and significantly increases the search time. Therefore, when two circles do not intersect due to case 1, we enlarge both of the circles by an equal amount so that they intersect. Likewise, if one is contained in the other, the inner circle is extended. These two cases are illustrated in Figure 4 a,b, respectively. When either case is encountered, the individual is penalized by the amount proportional to the total expansion of both circles. This process is summarized in Algorithm 3.


**Algorithm 3:** A function for calculating the intersection points**Function** IntersectionPoints (*L̂_i_,C_ik_, C_im_*) **begin** **if**
*C_ik_* ∩ *C_im_* ≠ Ø **then**  **return**
*C_ik_* ∩ *C_im_*; **end** **else if** ∥*l̂_ik_* − *l̂_im_*∥> *d_tk_* + *d_tm_*
**then**  // *C_ik_* and *C_im_* are separate  Δ*e =*∥*l̂_ik_* − *l̂_im_*∥ − (*d_tk_* + *d_tm_*);  punish *L̂_i_* by Δ*e*;  **return**
*(C_ik_* + Δ*e*/2) ∩ (*C_im_* + Δ*e*/2); **end** **else if** ∥*l̂_ik_* − *l̂_im_*∥ < |*d_tk_* − *d_tm_*| **then**  // Once circle is inside the other  Δe = |*d_tk_* − *d_tm_*| − ∥*l̂_ik_* − *l̂_im_*∥;  punish *L̂_i_* by Δ*e*;  **if**
*d_ik_* < *d_im_*
**then**   **return** (*C_ik_* +Δ*e*) ∩ *C_im_*;  **else**   **return** (*C_im_* +Δ*e*) ∩ *C_ik_*;  **end** **end****end**

**Figure 5.**
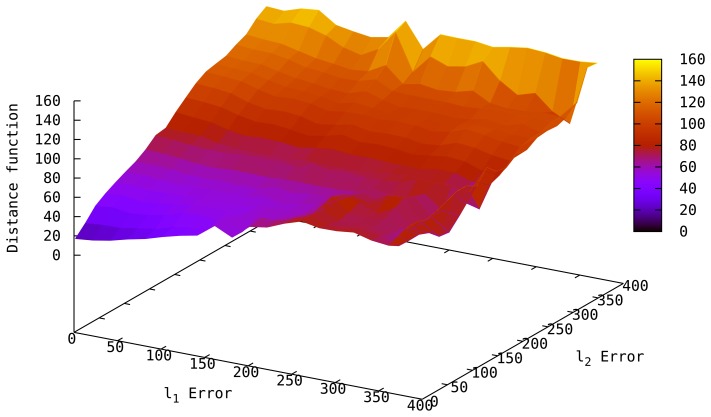
*Distance vs.* localization error in two sensors in a 4-sensor network.

Figure 5 shows the behavior of the *Distance* function with varying localization error in two sensors of a 4-sensor network when the remaining two sensors have perfect predictions. We observe the *Distance* function can correctly assess the quality of *L̂_i_*, such that, as the estimations deviate from the actual locations, the *Distance* of *L̂_i_* increases. This behavior also allows us to detect sensor displacements after localization by tracking abrupt changes in the *Distance* of the current topology (see Section 5 for details of displacement handling). Note that the distance of *L̂_i_* may not be 0 even if *L̂_i_* = *L* due to measurement errors; however, it will be smaller than most of the candidates.

**Figure 6.**
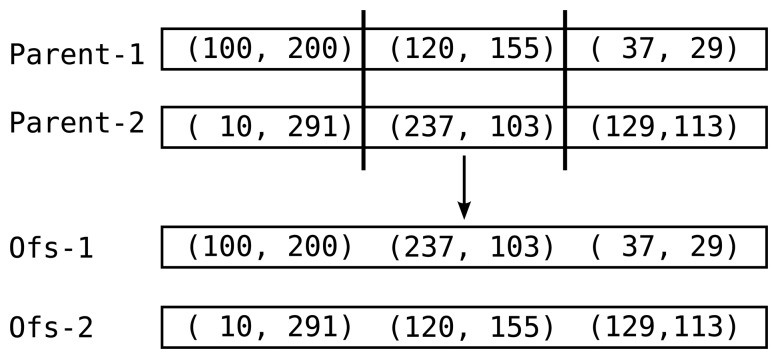
The crossover operator works on two parents to generate two offsprings.

**Step 2. Crossover:** After the quality estimation, current population, *G* is sorted according to the *Distance* values of individuals. In addition, a new generation, *G′* is created and initialized as an empty set. Pairs of individuals are then selected from G according to ranking selection that biases towards better individuals [30]. Each pair, with a probability of *R_x_*, goes through the crossover operator. This operator randomly selects the location estimation for one sensor from one of the parents to create two offsprings (Figure 6). These offsprings are then added to the next generation *G′*. With a probability of 1 − *R_x_*, input pairs are added to *G′* without any changes. This crossover operation aims to collect good genes, in the sense of improving per-sensor estimations, for the individuals to help convergence to the best solution around the discovered areas of the search space [31]. The selection and pairing process continues as long as |*G′*| < *z*.

**Step 3. Mutation**: Each individual in *G′* is modified with a probability of *R_m_* by selecting a sensor and a coordinate axis randomly and replacing the selected value with a random value. Mutation operation prevents the search from getting stuck in a local optima by introducing new areas of exploration. The mutation rate, *R_m_*, is generally chosen to be very small to reduce randomness. With high mutation rates, the search becomes essentially random [31,32]. Note that, we also use *elitism, i.e.*, always keep copies of the top-ranked 3 individuals with no modification. Elitism ensures that high quality individuals survive and contribute to the next generations. Following the mutation operation, the new generation *G′* becomes the new current generation *G* and the algorithm repeats itself for *Max_gen_* generations [33].

## 5. Target Tracking and Displacement Handling

Having identified the positions of landmarks, we develop an algorithm, called *Self-configuring Target Tracking* (SeT), which keeps track of the location of a mobile target using distance measurements from landmarks to the target as well as monitor if some of the landmarks are displaced. The former is achieved by using trilateration to locate the target, while the latter is handled in the same way as SeLL.

In order to improve the accuracy and the robustness of tracking and to effectively handle noise-prone ultrasonic sensors, the redundancy in the number of landmarks is exploited. In particular, SeT uses *K* (>3) landmark sensors such that the target location is determined as the average of trilateration results of three-landmark combinations of the *K* landmarks as shown in Equation (1).

(1) $$\frac{\underset{s\in \left(\underset{3}{\left|{\hat{L}}_{i}\right|}\right)}{\text{∑}}Trilateration(s)}{\left|\left(\underset{3}{\left|{\hat{L}}_{i}\right|}\right)\right|}$$

Although SeT can simultaneously detect and report positions of multiple targets, it cannot track the identity of multiple humans in a robust manner. For example, when two residents stop in close proximity of each other, SeT cannot maintain identities, meaning that other identification mechanisms are necessary to achieve multi-target tracking. Hence, we primarily focus on tracking a single target and reserve the latter as a future work.

**Figure 7.**
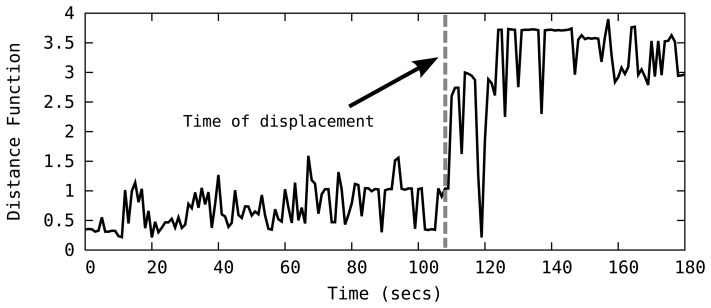
The *Distance* function abruptly changes upon a landmark displacement

To detect landmark displacements, SeT keeps track of the *Distance* function as part of the tracking process, looking for fluctuations in its value, as a sudden increase in the *Distance* suggests a displacement of landmarks. For instance, Figure 7 plots the *Distance* function over time in a four-node topology, indicating one of the landmarks in the network is displaced at time t = 108. Clearly, the occurrence of *displacement event* is immediately reflected on the *Distance* function as it suddenly starts producing large values. When the system detects a displacement event, it starts a *recovery* phase that detects the faulty landmark and attempts to reconstruct the network topology.

We take advantage of the redundant number of landmarks, e.g., *K* = 4, to identify which of the landmarks has been displaced. For this purpose, we define a *Distance*3 function that computes the distance between the point estimations for the target locations using 3 columns in the 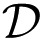 matrix. The *Distance*3 function is essentially the *Distance* function working only on 3 landmarks, and hence, equivalent to calculating the diameter of the prediction triangle for trilateration.

**Figure 8.**
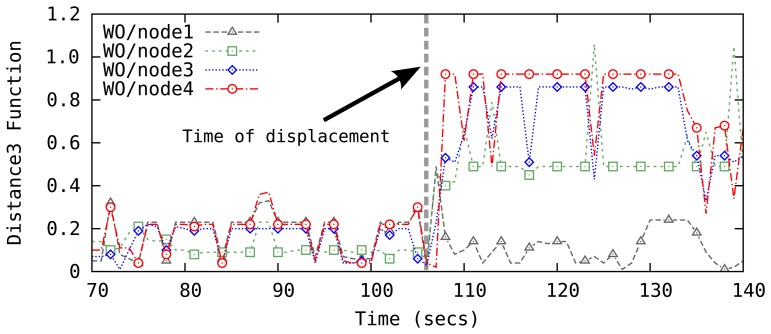
The *Distance* function for 3 nodes to detect the displaced node in case of a single node movement: only the *Distance*3 function without node1 (WO/node1) does not show a jump in its value.

We only consider a single landmark displacement assuming that it is unlikely that multiple landmarks are relocated within a short time window. When a displacement event is detected, the system with 4 landmarks forms four sets of 3 landmarks by removing one landmark from the input and calculates the *Distance*3 for each of the landmark set. If one of the landmark sets yields a significantly lower *Distance*3 value, the landmark left out for the calculation is determined to be the displaced one and a quick recovery process is initiated. Figure 8 illustrates a case in which node1 is displaced at time t = 108. In this figure, the change in the *Distance*3 functions for the three-landmark sets that include node1, *i.e.*, WO/node2–4, show a clear increase in the estimation error. In the recovery phase, a brute-force search for the current location of the displaced node is carried out. Due to low sensing range of ultrasonic ping sensors, this search is fast while the results are very accurate.

Tracking multiple objects requires modification to the SeT algorithm. First of all, an additional parameter, called an *expected diameter*, can be used in order to trigger the new multiple-object predictor. The algorithm can then check the diameter of a single object against the threshold and try to fit the data to a multi-object model in case exceeding the threshold. Unknowns of such a model are the number of objects, *N* and locations of the objects in the room, *P_i_*, 1 ≤ i ≤ *N*. These unknowns can be solved through a GA formulation where an individual is encoded as a tuple (*N, P_i_*) and the fitness being the distance between expected sensor data due to an individual and the actual sensor data. Note that stochastic minimization algorithms are observed to perform better with ultrasonic sensors due to various and frequent sources of errors.

## 6. Evaluation

**Figure 9.**
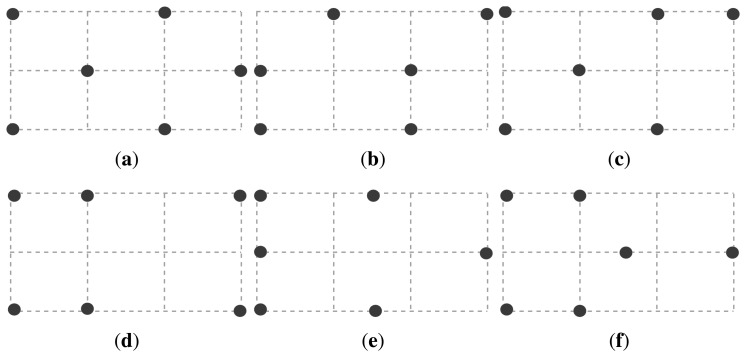
Six different topologies under test. (**a**) Topology-1; (**b**) Topology-2; (**c**) Topology-3; (**d**) Topology-4; (**e**) Topology-5; (**f**) Topology-6.

We evaluated our GA-based self-configuring localization protocol on a six-node testbed deployed within a 6 m^2^ room. As shown in Figure 9, a total of six different topologies were tested. We marked certain points on the ground as *checkpoints*. These checkpoints correspond to the intersection points of the dotted lines in Figure 9, in which, each dotted square measures 1 × 1m^2^. Sensor nodes are placed on these checkpoints and on the ground for ease of reconstructing the experiments, but we observe that slight differences in height from the ground do not have a significant impact on the performance of the algorithm. Sensors measure their distances to the object in a round robin fashion and wait for 0.5 s before each measurement. A participant walks between the checkpoints with no predetermined path while we record the time and the checkpoint location when the participant arrives at a checkpoint. Hence, we have the ground truth for the path taken by the participant. At some point in the experiment, we move one sensor node to an arbitrary, non-occupied checkpoint to mimic node displacement. A summary of the experimental setup and parameters for the GA are given in Table 1.

The experimental results are summarized in Table 2. To evaluate the performance of the proposed algorithm, we compute the accuracy of determining the locations of landmarks, quantified by a localization error, *ε_L_*, according to Equation (2), in which *L* refers to the actual locations of landmarks, and *l_k_* and *l̂_k_* are the actual and estimated location of a landmark, respectively.

(2) $$\varepsilon_{L}=\underset{l_{k}\in L}{\text{∑}}∥l_{k}-{\hat{l}}_{k}∥$$

Equation (2) computes the total of distances between actual and estimated locations of all of the landmarks in the topology. We also calculate the maximum tracking error, *ε_T_MAX__* and the average tracking error, *ε_T_AVG__* . *ε_T_MAX__* is the maximum distance encountered between the actual and estimated locations of the mobile object throughout the experiments, and *ε_T_AVG__* is the average of these errors reported with the 90 percentile range. Moreover, we report the time the system needs to infer a node displacement as the displacement detection latency, *t_D_*. In our experiments, we only tested a single node displacement and we claim this is a common case since it is unlikely that multiple nodes are displaced as noted earlier. We also report the time, *t_R_* for recovering from a displacement. We executed our algorithm on the collected data offline so that we were able to run the algorithm multiple times on the same data, however, the system is designed to run in real-time. We report the average of 10 runs for each dataset.

As summarized in Table 2, we observe, on average, 10 cm landmark localization error. *ε_L_* is relatively smaller for topologies that cover the circumference of the observed environment (Topology-1,4,5) with less than 10 cm average error for these topologies. Independent of the topology, the time to discover the locations of the landmarks during the self-configuration phase is about 6 min. This is expected since the execution flow is not heavily dependent on topological structure. Slight changes in the self configuration time are due to number of infeasible solutions generated during execution, which results in additional computations.

Maximum tracking error, *ε_T_MAX__* varies across topologies and it also depends on the path followed by the participant. Since we did not have a fixed path before the experiment, we cannot reliably evaluate the effect of topological formations on the tracking error. Generally, a higher landmark localization error yields higher tracking errors, and maximum observed tracking error is between 16 to 30 cm. In Table 2, we also report the average tracking error including 90 percentile error ranges. On the average, we can track a mobile target with 20 cm accuracy in a 6 m^2^ room.

**Figure 10.**
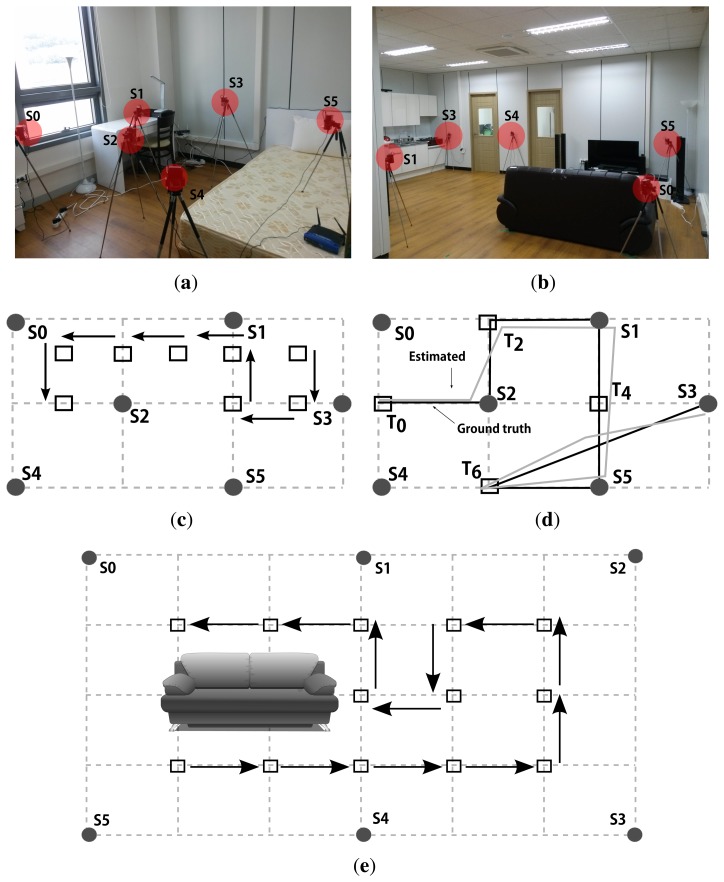
Tracking tests on two real testbeds of different sizes where *S_i_*s indicate sensors. (**a**) Picture of 2 × 3 m^2^ testbed; (**b**) Picture of 4 × 6 m^2^ testbed; (**c**) Tracking test 1 in a 2 × 3 m^2^ testbed; (**d**) Tracking test 2 in a 2 × 3 m^2^ testbed; (**e**) Tracking test 3 in a 4 × 6 m^2^ testbed.

We further evaluated the tracking performance of our protocol under two topologies of different sizes, *i.e.*, 2 × 3 m^2^ and 4 × 6 m^2^, as shown in Figure 10. We first measured the tracking performance for the testbed in Figure10c. We report 90% confidence level for 10 runs. The localization error, *ε_L_* is 7.16 cm while the self-configuration time, *t_SC_* is 4.03 min. The 90 percentile maximum tracking error, *ε_T_MAX__* is 21.27 cm and the average tracking error, *ε_T_AVG__* is 11.71 cm. We also conducted a tracking test in the same testbed, and present an example tracking estimation along with the ground truth in Figure10d. Note that the black path is an approximation of the ground truth while the gray path is an estimation by the system.

We then measured the tracking performance for a larger testbed shown in Figure 10e, which is a sitting room setup with a sofa in the middle. We ran 10 experiments to report the following results. *ε_L_* and *t_SC_* are 17.53 cm and 8.23 min, respectively. *ε_T_MAX__* is 24.46 cm while an absolute maximum tracking error over all 10 runs is 36.28 cm. Finally, *ε_T_AVG__* is 18.43 cm. We believe these results are satisfactory, although there is also a large room for improvement since we are putting no efforts in post processing, e.g., smoothing, the tracks generated by our system.

**Figure 11.**
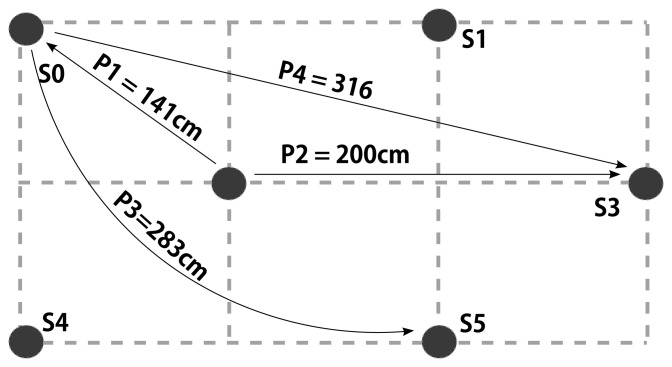
Displacement patterns for measuring displacement detection performance.

We finally evaluated the performance of our displacement detection and topology correction algorithm according to the pattern given in Figure 11. The experiments are carried out by first recording sensor data from all sensors in Figure 11, and later selectively suppressing the sensor at the target location until a predetermined time of displacement. We then suppress the source node and start streaming the target node creating a virtual displacement. This method allows us the speedup the process of analysis.

The results of the experiments are summarized in Table 3. We observe that *ε_T_MAX__* is inclined to increase with increasing displacement. This result is expected due to larger errors during the reconfiguration phase. Surprisingly, the magnitude of displacement does not seem to affect the detection latency. Hence, as long as there is a *significant* discrepancy between estimated layout and sensor data, the system can infer a displacement. The latency of detection is approximately 30 s and we do not expect to see lower values with our hardware because the system should be carefully designed in order not to enter into the reconfiguration phase frequently. Note that even multi-path effects may cause such discrepancies. Nevertheless, this latency can still be further reduced using higher quality sensors. Similarly, the recovery time, *t_R_*, after a displacement does not seem to depend on the magnitude of the displacement. Rather, we consider that the time required for reconfiguration is a function of the resulting topology. We observe that within 5 s of the detection, the system reconstructs the model.

## 7. Conclusions and Future Work

In this paper, we presented a self-configuration protocol for device-free target tracking systems. The proposed protocol first maps the geographic topology of a network of low-cost ultrasound sensors without using nodes with known locations, then proceeds to track humans using the same hardware. We also developed a lightweight sensor displacement scheme that can detect any changes in the topology in less than a minute and initiate a quick recovery phase to generate a new map within seconds. Finally, we demonstrated via experiments that our proposed system can produce highly accurate results over a broad range of network layouts. In the future, we will extend our tracking system to add the capability of tracking multiple targets.

## Figures and Tables

**Table 1.** Experimental setup and Genetic Algorithm (GA) parameters.

**Parameter**	**Value**
Number of generations	400
Population size	100
Mutation rate (*R_m_*)	0.1
Crossover rate (*R_x_*)	0.6
Number of elites	3
Area size	3 × 2 (m^2^)
Number of sensors	6
Sensing delay	0.5 (s)

**Table 2.** Experiment results per topology: localization error (*ε_L_*), self-configuration time (*t_SC_* ), maximum and average tracking errors (*ε_TMAX_* , *ε_TAVG_* ), displacement detection latency (*t_D_*), and time taken to recover from 1-landmark displacement (*t_R_*).

**Topology**	***ε****_L_* **(cm)**	***t****_SC_* **(min)**	***ε****_T_MAX__* **(cm)**	***ε****_T_AVG__* **(cm)**	***t****_D_* **(s)**	***t****_R_* **(s)**
**Topology-1**	8.23	5.69	19.62	12.47 ± 3.56	24.64	3.14
**Topology-2**	12.87	5.71	29.50	16.39 ± 7.01	36.71	3.92
**Topology-3**	13.60	5.32	25.21	14.21 ± 6.22	43.37	3.11
**Topology-4**	5.41	5.43	22.09	9.57 ± 8.19	21.09	3.49
**Topology-5**	9.21	4.98	23.42	12.13 ± 9.08	29.50	2.91
**Topology-6**	14.40	6.07	16.01	6.08 ± 7.56	33.99	3.06

**Table 3.** Displacement performance results for scenarios in Figure 11: Maximum and average tracking errors (*ε_TMAX_* , *ε_TAVG_* ), displacement detection latency (*t_D_*), and time taken to recover from 1-landmark displacement (*t_R_*)

**Scenario**	**Distance (cm)**	***ε****_T_MAX__* **(cm)**	***ε****_T_AVG__* **(cm)**	***t****_D_* **(s)**	***t****_R_* **(s)**
**P1**	141	17.53	8.50 ± 3.17	27.47	3.24
**P2**	200	16.01	7.23 ± 2.52	30.34	2.19
**P3**	283	23.38	11.19 ± 2.46	32.45	4.54
**P4**	316	29.14	13.03 ± 2.14	30.32	3.37
